# LymphUs: A multicenter open-access database of lymph node ultrasound images in patients with papillary thyroid carcinoma for clinical and artificial intelligence research

**DOI:** 10.1016/j.dib.2026.112694

**Published:** 2026-03-17

**Authors:** Afshin Mohammadi, Alisa Mohebbi, Mohammad Mirza-Aghazadeh-Attari, Saeed Mohammadzadeh, U Rajendra Acharya, Ru-San Tan, Massimo Salvi, Sepideh Hatamikia, Ali Abbasian Ardakani

**Affiliations:** a Department of Radiology, Faculty of Medicine, Urmia University of Medical Science, Urmia, Iran; b Clinical AI-Research in Omics and Medical Data Science (CAROM) group, Department of Medicine, Faculty of Medicine and Dentistry, Danube Private University, Krems an der Donau, Austria; c Division of Vascular and Interventional Radiology, Russell H. Morgan Department of Radiology and Radiological Sciences, Johns Hopkins University, Baltimore, MD, USA; d Universal Scientific Education and Research Network (USERN), Tehran, Iran; e School of Medicine, Tehran University of Medical Sciences, Tehran, Iran; f School of Mathematics, Physics and Computing, University of Southern Queensland, Springfield, Queensland, Australia; g Centre for Health Research, University of Southern Queensland, Springfield, Queensland, Australia; h National Heart Centre Singapore, Singapore; i Duke-NUS Medical School, Singapore; j Biolab, PolitoBIOMedLab, Department of Electronics and Telecommunications, Politecnico di Torino, Turin, Italy; k Austrian Center for Medical Innovation and Technology (ACMIT), Wiener Neustadt, Austria; l Department of Radiology Technology, School of Allied Medical Sciences, Shahid Beheshti University of Medical Sciences, Tehran, Iran

**Keywords:** Database, Lymph nodes, Machine learning, Thyroid cancer, papillary, Ultrasonography, Deep Learning

## Abstract

Approximately 30–50% of Papillary thyroid carcinoma (PTC) patients develop cervical lymph nodes (LNs) metastasis, significantly increasing the risk of disease recurrence and impacting long-term outcomes. We introduced an open-access multicenter lymph node ultrasound image database (LymphUs) specifically designed to advance research in LN assessment for PTC. Ultrasound imaging was performed on PTC patients at two independent clinical centers using standardized acquisition protocols. Experienced radiologists at each center documented sixteen semantic features for each LN. All LNs were annotated with segmentation masks serving as ground truth, and classification into benign or malignant categories was confirmed by fine needle aspiration biopsy results. The LymphUs comprises ultrasound images with segmentation masks from 338 PTC patients with suspected LN metastasis, divided into two center-specific cohorts: 180 patients (81 malignant, 99 benign) and 158 patients (82 malignant, 76 benign). The complete dataset, including semantic features and expert annotations, is freely accessible for research purposes. The LymphUs bridges a critical gap in medical imaging resources by providing a large-scale, multicenter ultrasound database for cervical LN assessment in PTC, supporting diagnostic algorithms, standardized reporting systems, and artificial intelligence applications to enhance preoperative LN staging and treatment planning.

## Graphical abstract

**Figure:**
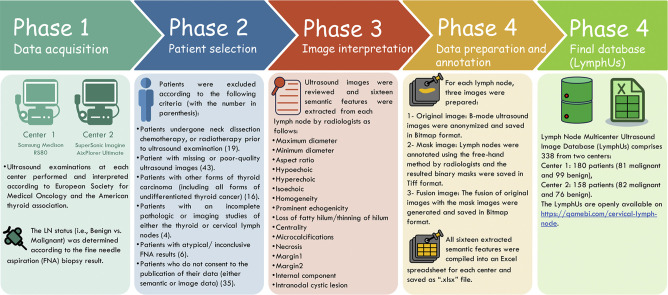

Specifications Table

**Table:** 

Subject	Health Sciences, Medical Sciences & Pharmacology
Specific subject area	Oncology, Radiology
Type of data	Image, Excel spreadsheet
Data collection	Patients with papillary thyroid carcinoma and suspected cervical lymph node metastasis were evaluated at two independent clinical centers with retrospective data extraction from electronic medical records and radiological PACS systems.
Data source location	Institution: Shahid Beheshti University of Medical Sciences, Tehran, Iran
Data accessibility	Repository name: QAMEBI Direct URL to data: https://qamebi.com/cervical-lymph-node Folder/File: Center 1.zip and Center 2.zip containing ultrasound images with segmentation masks, fusion images, and comprehensive imaging features in Excel format.
Related research article	Abbasian Ardakani A, Mohammadi A, Mirza-Aghazadeh-Attari M, Faeghi F, Vogl TJ, Acharya UR. Diagnosis of Metastatic Lymph Nodes in Patients With Papillary Thyroid Cancer: A Comparative Multi-Center Study of Semantic Features and Deep Learning-Based Models. Journal of ultrasound in medicine: official journal of the American Institute of Ultrasound in Medicine. 2023;42(6):1211–1221. https://doi.org/10.1002/jum.16131 [1]

## 1 Value of the Data

• First multicenter open-access ultrasound database — To our knowledge, this is the first publicly available dataset combining semantically annotated lymph node ultrasound images from two independent clinical centers with comprehensive feature documentation and confirmed pathological validation for PTC research. Specific applications include development of diagnostic AI models, automated segmentation networks, inter-observer agreement studies, teleradiology algorithms, educational platforms, and individual-participant data (IPD) meta-analyses.
• Standardized clinical methodology – The dataset includes rigorous ultrasound protocols adhering to guidelines with experienced radiologists (14 and 23 years of experience) at each center, ensuring high clinical relevance and applicability to routine diagnostic practice.
• Rich semantic annotation – Each lymph node image is accompanied by annotated imaging features (morphology, echogenicity, borders, internal composition) enabling detailed analysis of imaging characteristics and development of classification algorithms for benign versus malignant differentiation.
• Balanced multicenter design – With nearly equal distribution of benign (175 nodes) and malignant (163 nodes) cases across two distinct centers, the dataset minimizes class imbalance bias and enables both internal and external validation of artificial intelligence models. The LymphUs database provides 80% statistical power to detect clinically meaningful AUC differences of 0.10 between competing AI models for lymph node classification (α=0.05), addressing key limitations of existing single-center datasets while enabling robust multicenter validation.
• Ground truth validation – All lymph nodes have histopathological or cytopathological confirmation biopsy, providing reliable gold-standard labels for supervised machine learning and deep learning algorithm training and testing.
• Segmentation masks and fusion images – The inclusion of binary segmentation masks (.tif format) and overlay fusion images facilitates rapid implementation of computer vision algorithms and reduces manual annotation burden for researchers developing automated segmentation methods.
• Applications across research domains – The dataset supports development and validation of machine learning classification models, deep learning segmentation networks, radiomics analysis, inter-observer agreement assessment, diagnostic accuracy studies, and education of radiology trainees.

## 2 Background

Papillary thyroid carcinoma (PTC) spreads predominantly via the lymphatic system, with 30–50% of PTC patients having cervical lymph node metastasis (CLNM) at presentation [2]. The presence of CLNM significantly increases the risk of local recurrence and distant metastasis, leading to worse long-term outcomes and reduced overall survival [3].

Ultrasound has emerged as the primary diagnostic method for thyroid cancer and is now recommended as the preferred modality for CLNM assessment in PTC patients [3]. Although preoperative ultrasound demonstrates high sensitivity (85–93%) and moderate specificity (65–80%), differentiating benign from malignant LNs based solely on ultrasound parameters remains challenging, highlighting the need for robust diagnostic criteria [4,5].

Recent advances in artificial intelligence (AI) methods have shown promising results for assessing LNs in PTC patients [1,6,7]. However, AI model generalizability is limited by domain shift, which occurs when external validation datasets are acquired under different conditions or with different devices [8,9]. Current publicly available LN imaging databases have significant limitations: most originate from single institutions, focus on CT rather than ultrasound imaging, and lack comprehensive annotations. To address these gaps, we have developed the LymphUs database, an open-access repository of ultrasound images from PTC patients with suspected lymph node metastasis.

## 3 Data Description

### 3.1 Patient characteristics

The LymphUs database comprises ultrasound images and comprehensive annotations from 338 patients with histologically confirmed papillary thyroid carcinoma (PTC) and suspected lymph node (LN) metastasis. A key strength of this database is its multicenter design, with data collected from two independent clinical centers using different ultrasound equipment. This design specifically addresses a significant limitation of most existing radiological databases, which typically include data from only a single center, restricting their utility to either training with internal validation or external validation alone.

The database is organized into two distinct sections based on the contributing centers:

• Center 1: 180 patients (81 with metastatic/malignant LNs and 99 with reactive/benign LNs)
• Center 2: 158 patients (82 with metastatic/malignant LNs and 76 with reactive/benign LNs)

**Fig. 1:**
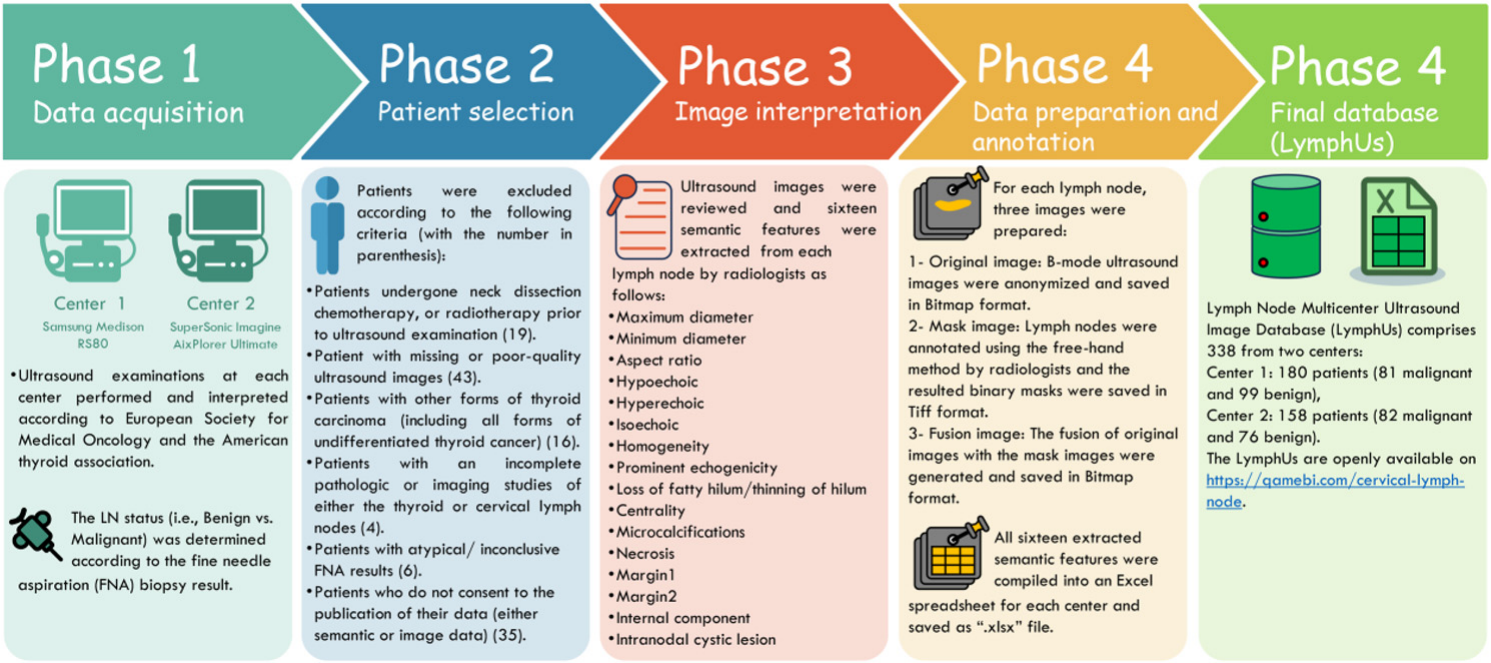
Overall phases involved in this study to generate the LymphUs.

This structure enables researchers to use Center 1 data for model training and internal validation while reserving Center 2 data for external validation, or vice versa, facilitating robust model development and testing. The nearly balanced distribution of benign and malignant cases enhances the database's value for developing classification algorithms, as it reduces the risk of class imbalance bias during model training. The overall recruitment flowchart is depicted in Fig. 1.

**Fig. 2:**
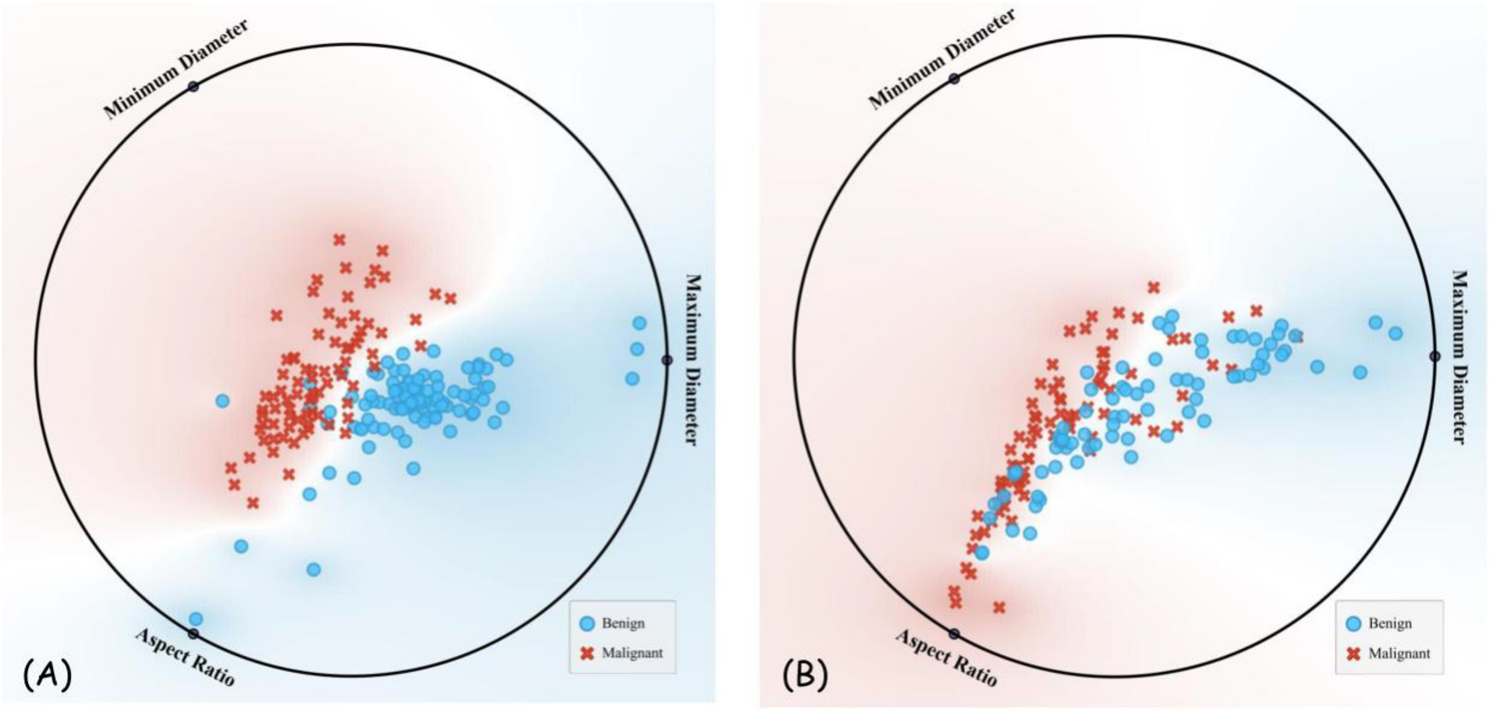
RadViz plot of quantitative features distribution (maximum and minimum diameters, and aspect ratio of lymph nodes) for centers 1 (A), and 2 (B).

For each patient, a comprehensive set of sixteen imaging features was documented, including quantitative measurements (maximum diameter, minimum diameter, aspect ratio) and qualitative characteristics (echogenicity patterns, structural features, and border characteristics). These features were systematically evaluated by experienced radiologists and compiled into structured Excel spreadsheets that are accessible through the database website. Table 1 presents the center-wise distribution of these imaging features, highlighting differences and similarities between malignant and benign LNs across both centers. Fig. 2 shows RadViz plots of quantitative feature distribution stratified by center and LN status.

**Table 1:** The distribution of sixteen lymph nodes features among the two centers.

Imaging characteristic		Center 1		Center 2	
		Malignant (*n* = 81)	Reactive (*n* = 99)	Malignant (*n* = 82)	Reactive (*n* = 76)
Maximum diameter	mm	16.1 (CI = 13.9 to 18.2)	13.0 (CI = 10.4 to 15.5)	14.4 (CI = 12.7 to 16.1)	14.3 (CI = 13.1 to 15.4)
Minimum diameter	mm	10.6 (CI = 9.0 to 12.3)	3.4 (CI = 2.7 to 4.0)	8.9 (CI = 7.8 to 10.0)	5.7 (CI = 5.2 to 6.2)
Aspect ratio		0.68 (0.64 to 0.72)	0.34 (0.26 to 0.42)	0.64 (0.60 to 0.68)	0.42 (0.39 to 0.46)
Hypoechoic	Yes	74 (91.3%)	98 (98.9%)	69 (84.1%)	76 (100%)
Hyperechoic	Yes	46 (56.7%)	1 (1.0%)	35 (42.6%)	0 (0%)
Isoechoic	Yes	5 (6.1%)	1 (1.0%)	13 (15.8%)	0 (0%)
Homogeneity	Heterogenous	45 (55.5%)	0 (0%)	35 (42.6%)	0 (0%)
Prominent echogenicity (For heterogenous nodes)	Hyperechoic	22 (27.1%)	98 (98.9%)	1 (1.2%)	0 (0%)
	Hypoechoic	54 (66.6%)	0 (0%)	68 (82.9%)	76 (100%)
Loss of fatty hilum/thinning of hilum	Present	81 (100%)	18 (18.1%)	78 (95.1%)	9 (11.8%)
Centrality (Eccentric vs. Concentric thickening of cortex)	Eccentric thickening of cortex	0 (0%)	0 (0%)	64 (78.0)	3 (3.9%)
Microcalcifications	Present	45 (55.5%)	0 (0%)	40 (48.7%)	0 (0%)
Necrosis	Cystic	5 (6.1%)	0 (0%)	7 (8.5%)	0 (0%)
	Coagulative	0 (0%)	0 (0%)	5 (6.0%)	1 (1.3%)
Margin1	Irregular	41 (50.6%)	0 (0%)	26 (31.7%)	2 (2.6%)
Margin2	Ill-defined	24 (29.6%)	0 (0%)	0 (0%)	0 (0%)
Internal component	Mixed	5 (6.1%)	0 (0%)	9 (10.9%)	0 (0%)
Intranodal cystic lesion	Present	3 (3.7%)	0 (0%)	12 (14.6%)	1 (1.3%)

### 3.2 Image annotation & segmentation

Data files containing all records referred to in this paper as ``LymphUs'' can be found at https://qamebi.com/cervical-lymph-node. The complete database is approximately 50 MB in size and is organized by clinical center. Center 1.zip contains all data from the first clinical center, while Center 2.zip contains all data from the second clinical center. Each zip file contains three main components: an Excel file with comprehensive imaging findings as described in section 4.2, a Benign folder containing all images of patients with reactive/benign lymph nodes, and a Malignant folder containing all images of patients with metastatic/malignant lymph nodes.

Ultrasound Examinations did utilize high-frequency linear transducers (Center 1: Samsung RS80, 5–12 MHz L5–12/60; Center 2: AixPlorer Ultimate, 4–15 MHz SL15–4). Standardized settings included multiple focal zones (2–3 cm depth), TGC-adjusted gain (50–60%), dynamic range 60–80 dB, and 20–30 fps frame rates. All cervical LN levels imaged in transverse/longitudinal planes with cine-loop capture, per ESMO/ATA guidelines. All ultrasound images were originally exported from the machines and stored in BMP format. Before release, the images were anonymized, and any on-image identifiers (embedded text/overlays) were removed by cropping the images in MATLAB software (version 2024a), so that no patient-related information remained in the shared files. To facilitate reproducible AI preprocessing, we now provide key image-level metadata. All ultrasound images are grayscale and stored as 8-bit images. The pixel matrix size varies across cases and between centers: for Center 1, image size ranges from 248×226 to 570×570 pixels (mean 407×406), while for Center 2 it ranges from 290×290 to 826×724 pixels (mean 468×455).

**Fig. 3:**
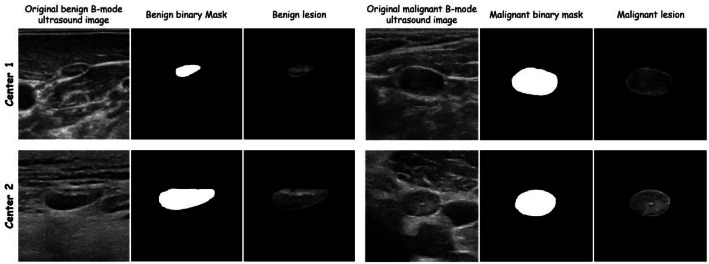
Sample images of benign (First row) and malignant (second row) lymph nodes with the corresponding masks and fused images for both centers.

The Excel file includes an ``Image name'' column that provides a direct link to the corresponding ultrasound images. For each patient case, three types of image files are provided. Original ultrasound images are named with a consecutive number followed by the group name and ``Image.bmp'' (e.g., ``1 Malignant Image.bmp''). Segmentation mask images, which serve as ground truth annotations, are named with a consecutive number followed by the group name and ``Mask.tif'' (e.g., ``1 Malignant Mask.tif''). Fusion images, which are overlays of the original and mask images, are named with a consecutive number followed by the group name and ``Lesion.bmp'' (e.g., ``1 Malignant Lesion.bmp''). Representative PTC examples from both centers, including those involving malignant and reactive LNs, are shown in Fig. 3.

Table 2 presents the primary open datasets for LN imaging [[10], [11], [12], [13], [14]]. Most of these datasets originate from single institutions and tend to have moderately smaller sample sizes, often lacking mask images. The landscape of publicly available datasets highlights a disparity in imaging modalities, with many existing datasets derived from CT imaging, whereas ultrasound-based datasets are notably limited. This underscores a pressing need for more ultrasound-focused resources to enhance both research and clinical applications in this field. One notable advantage of LymphUs compared to other datasets is its homogenous, disease-specific nature, concentrating solely on PTC, among the most prevalent malignancies. This focused approach ensures a consistent and well-defined dataset that is particularly beneficial for developing and validating diagnostic and predictive models tailored to this specific cancer.

**Table 2:** Examples of open-access datasets providing lymph node imaging.

Dataset	Country	Latest version (year)	Number of patients	Presented condition	Imaging modality	Center status	Is masking performed?
Roth et al. [14]	USA	2014	176	Non-cancerous lymphadenopathy	CT	Single center	No
Maroongroge et al. [12]	USA	2024	46	Head & Neck cancers	CT	Single center	No
Luo et al. [11]	China	2024	262	Head & Neck cancers	CT	Multicenter	No
Mehrtash et al. [13]	USA	2024	513	Various cancers	CT	Multicenter	Yes
Ludwig et al. [10]	China	2022	287	Head & Neck cancers	MRI/CT, PET-CT	Single center	No
LymphUs dataset (proposed)	Iran	2025	338	Head & Neck cancers	Ultrasound	Multicenter	Yes

The LymphUs offers a wide range of potential applications spanning both clinical diagnostics and cutting-edge research endeavors. Its comprehensive nature and standardized format make it suited to advance the field of LN assessment, particularly in the context of PTC:

• **Diagnostic AI models:** The LymphUs can be utilized to develop advanced machine learning prediction models. These models can facilitate the automated classification of lymph node malignancy, potentially enhancing diagnostic accuracy and efficiency. With its relatively large sample size and multicenter nature, the database supports both internal and external validation of the models, ensuring their robustness and applicability across diverse patient populations and clinical environments. Additionally, rigorous comparative studies can be conducted to assess radiologists' performance with and without the assistance of AI models, offering valuable insights into the added benefits of artificial intelligence in clinical decision-making.
• **Segmentation using AI models:** In addition to diagnostic classification, the LymphUs promotes the development of deep-learning models designed for precise lymph node segmentation. Accurate segmentation is essential for effective quantitative image analysis and is vital in identifying subtle features that may suggest malignancy. These segmentation models can be incorporated into clinical workflows to enhance the lymph node evaluation process, minimizing inter-observer variability and ensuring more consistent measurements.
• **Agreement assurance:** The inherent variability in interpreting ultrasound images underscores the need for robust methods to assess the consistency and reproducibility of findings. The LymphUs serves as a standardized reference for evaluating the performance of various radiologists, allowing for the quantification of inter-observer agreement in ultrasound examination findings and reports. This capability is essential for quality control and helps identify areas where additional radiological training or standardization may be necessary.
• **Clinical research:** The data contained within the LymphUs can be leveraged to address critical clinical questions associated with LN metastasis in PTC. In particular, the database can be employed to develop and validate risk stratification regression models that incorporate clinical and imaging features to predict the likelihood (i.e., probability) of lymph node involvement. These models can aid in clinical decision-making regarding the necessity for additional diagnostic evaluations or therapeutic interventions. Furthermore, the LymphUs may be a valuable resource for exploring fundamental clinical inquiries, such as the optimal role of LN ultrasound in detecting, localizing, and characterizing metastatic disease.
• **Teleradiology and Remote Detection:** The growing demand for remote healthcare solutions highlights the need for advanced tools in telemedicine applications. The LymphUs can be utilized to create algorithms for the remote assessment of lymph nodes through ultrasound images, facilitating expert consultation and diagnosis in regions with limited access to specialized healthcare services. This capability can potential to enhance the timeliness and accessibility of care for patients in developing communities.
• **Educational purposes:** The LymphUs can be utilized as an invaluable resource for educational institutions aiming to improve the training of residents and fellows in ultrasound interpretation. By offering access to a comprehensive and well-characterized dataset of lymph node ultrasound images, the database facilitates hands-on learning. It promotes the development of expertise in distinguishing between reactive and metastatic lymph nodes. The diversity inherent in the LymphUs guarantees that trainees receive consistent, high-quality instruction, equipping them with challenges they may face in clinical practice.
• **Meta-analysis:** Acknowledging the limitations inherent in individual aggregated studies, the LymphUs is designed to be interoperable with similar databases that may emerge and be published in the future. By integrating the LymphUs with these complementary datasets through individual-participant data (IPD) meta-analysis, researchers can produce more robust evidence concerning the diagnostic efficacy of ultrasound in assessing lymph node malignancy in PTC patients. Such collaborative initiatives are crucial for progressing the field and establishing evidence-based guidelines for clinical practice.

## 4 Experimental Design, Materials and Methods

### 4.1 Recruitment & ethical approval

The LymphUs database was developed through retrospective collection of data from 338 patients with suspected thyroid cancer who had undergone ultrasound evaluation for enlarged cervical LNs at two independent centers. The Institutional Review Board approved the protocol for retrospective collection of medical information and images, and all study participants provided written informed consent. All patient information and images were anonymized prior to inclusion in the database. Inclusion criteria were: (1) cytological confirmation of primary PTC; (2) presence of one or more enlarged LN in the cervical region; and (3) completed preoperative cervical LN ultrasound examination.

To maximize the database's clinical representativeness, we selected only one LN per patient. This approach prevented potential bias from patients with extensive LN involvement who would otherwise contribute disproportionately to the dataset. The LN status (i.e., metastasis vs. reactive) was based on ultrasound-guided fine needle aspiration (FNA) biopsy results, providing cytological confirmation for all included cases. We excluded patients who had undergone neck dissection, chemotherapy, or radiotherapy prior to ultrasound examination, as well as those with missing or poor-quality ultrasound images. Patients with other forms of thyroid carcinoma, incomplete pathological or imaging examinations, and atypical/ inconclusive FNA results were excluded. In addition, patients who did not consent to the publication of their data (either semantic or image data) were not included in the database. Beyond these exclusions, we adopted an inclusive approach for patient enrollment to ensure the database reflects real-world clinical heterogeneity.

We included patients with distant metastases, varying differentiation rates (i.e., poor, moderate, well), bilateral PTC, background thyroid diseases (e.g., Hashimoto thyroiditis, chronic lymphocytic thyroiditis), central LN involvement, positive family history of PTC [15,16]. Since PTC can affect younger populations, we included appropriately consented patients under 18 years of age. Patients below 18 years provided assent, and written informed consent was obtained from their parents or legal guardians (Fig. 1). Our approach preserves the database’s diversity and helps preventing overfitting in future AI prediction models trained on or validated using LymphUs.

### 4.2 Image acquisition & interpretation

In this study, ultrasound imaging was performed using the Samsung Medison RS80 (Samsung Medison, Seoul, Korea) at Center 1 and the SuperSonic Imagine AixPlorer Ultimate (SuperSonic Imagine, Aix-en-Provence, France) at Center 2. Two experienced radiologists (with 14 and 23 years of experience) had separately performed ultrasound examination and FNA and interpreted the ultrasound findings at their respective centers. The radiologists were blinded to pathology results and clinical outcomes when interpreting the ultrasound findings. All examinations followed standardized protocols according to the European Society for Medical Oncology (ESMO) and the American Thyroid Association (ATA) guidelines [3,17].

For each examination, patients were positioned supine with their necks fully extended and exposed at the front. Using a linear probe, radiologists visualized all six lymph node regions of the neck from multiple views, correlating imaging findings with physical LN examinations. The thyroid lobes and surrounding anatomical structures (common carotid artery, recurrent laryngeal nerve, trachea, esophagus, and jugular vein) were also thoroughly assessed. To ensure precise correlation between imaging and cytology, the radiologists marked the skin corresponding to the target LN location prior to FNA. For each evaluated LN, radiologists documented a comprehensive set of sixteen standardized semantic features, organized into the following categories:

**Size and Shape Parameters:**

• Maximum diameter: This feature refers to the longest axis of the LN, measured explicitly as the height in millimeters (mm).
• Minimum diameter: The shortest axis of the LN, referred to as the width, is also measured in millimeters. This measurement complements the maximum diameter to provide a complete picture of the LN's dimensions.
• Aspect ratio: This feature is calculated as the ratio of the minimum diameter to the maximum diameter of the LN. It serves as an important indicator of the LN shape.

**Echogenicity Characteristics:**

• Hypoechoic: This binary feature indicates the presence or absence of any hypoechoic sections within the LN.
• Hyperechoic: Similar to hypoechoic, this feature denotes whether there are any hyperechoic sections present in the LN.
• Isoechoic: This feature assesses whether the entire LN appears isoechoic compared to surrounding tissues. Isoechoicity can complicate LN pathology detection, as it may mask underlying pathology.
• Homogeneity: This feature evaluates the internal echotexture of the LN based on echogenicity, indicating whether there is uniformity or variation within the LN's structure. A homogeneous appearance may suggest benign conditions, while heterogeneity could raise suspicion for malignancy.
• Prominent echogenicity (for heterogeneous nodes): In cases where LNs exhibit heterogeneous characteristics, this feature categorizes their most notable visual appearance as hypoechoic, hyperechoic, or isoechoic.

**Structural Features:**

• Loss of fatty hilum/thinning of hilum: This feature assesses whether there is an indentation or notch on the medial portion of the LN, which can indicate pathological involvement and loss of typical fatty architecture.
• Centrality (eccentric vs. concentric thickening of cortex): This feature differentiates between concentric cortex thickening—where there is uniform thickening around the entire circumference of the LN—and eccentric cortex thickening, where thickening occurs unevenly on one side.
• Microcalcifications: This binary feature indicates the presence or absence of small echogenic spots within the evaluated LN parenchyma.
• Necrosis: This feature evaluates whether there is evidence of coagulative necrosis (characterized by heterogeneous and hypoechoic areas), cystic necrosis (well-defined and anechoic areas), or an absence of necrosis.

**Border and Content Characteristics:**

• Margin1: This feature describes whether the margins of the LN are regular (smooth borders) or irregular (jagged borders).
• Margin2: Similar to Margin1, this feature assesses whether the margins are well-defined (clear borders) or ill-defined (indistinct margins).
• Internal component: This feature classifies the internal structure of the LN as solid (homogeneous and without visible fluid-filled areas) or mixed with cystic components (presence of fluid-filled cavities).
• Intranodal cystic lesion: This binary feature indicates whether anechoic fluid-filled cavities are present within the LN parenchyma.

## Limitations

The released database has some limitations that need to be addressed and improved in the future: (1) Other ultrasound methods, such as Doppler, elastography, and contrast-enhanced ultrasounds, may provide valuable additional information in assessing LN involvement and will be considered for inclusion in future version [[18], [19], [20]]; (2) Despite including metastatic (malignant) and reactive (benign) LNs, no normal LN was included due to ethical concerns about performing FNA on cervical regions without a clinical indication; (3) In some cases, arrow markings were inserted by radiologists during the examinations to localize the LN of interest. While these could potentially be confounding factors for AI models, they are present in both benign and malignant groups, suggesting that sophisticated algorithms would not consider these arrows as informative features for classification; (4) This database was utilized in our previous study [1], which established baseline performance metrics for LN classification. It should be noted that data from 35 patients included in our previous work were excluded from LymphUs as these patients did not consent to the publication of their data.

## Ethics Statement

Institutional Review Board (IRB) approval was obtained for this study by Research Ethics Committees of ViceChancellor in Research Affairs –vtool Shahid Beheshti University of Medical Sciences, Tehran, Iran: #IR.SBMU.RETECH.REC.1400.696.

## CRediT Author Statement

**Afshin Mohammadi:** Methodology, Formal analysis, Data curation, Validation, Investigation, Visualization, Writing – Original Draft. Writing – review and editing. **Alisa Mohebbi:** Formal analysis, Data curation, Validation, Investigation, Visualization, Writing – Original Draft. Writing – review and editing. **Mohammad Mirza-Aghazadeh-Attari:** Formal analysis, Validation, Investigation, Visualization, Writing – Original Draft. Writing – review and editing. **Saeed Mohammadzadeh:** Formal analysis, Validation, Investigation, Visualization, Writing – Original Draft. Writing – review and editing. **U Rajendra Acharya:** Methodology, Validation, Visualization, Writing – Original Draft. Writing – review and editing. **Ru-San Tan:** Validation, Visualization, Writing – Original Draft. Writing – review and editing. **Massimo Salvi:** Validation, Visualization, Writing – Original Draft. Writing – review and editing. **Sepideh Hatamikia:** Methodology, Validation, Visualization, Writing – Original Draft. Writing – review and editing. **Ali Abbasian Ardakani:** Methodology, Data curation, Supervision, Resources, Validation, Visualization, Writing – Original Draft. Writing – review and editing.

## Data Availability

LymphUs (Original data)https://qamebi.com/cervical-lymph-node/
